# Structural neuroimaging of social cognition in progressive non-fluent aphasia and behavioral variant of frontotemporal dementia

**DOI:** 10.3389/fnhum.2013.00467

**Published:** 2013-08-16

**Authors:** Blas Couto, Facundo Manes, Patricia Montañés, Diana Matallana, Pablo Reyes, Marcela Velasquez, Adrián Yoris, Sandra Baez, Agustin Ibáñez

**Affiliations:** ^1^Laboratory of Experimental Psychology and Neuroscience (LPEN), Institute of Cognitive Neurology (INECO), Favaloro UniversityBuenos Aires, Argentina; ^2^Institute of Neuroscience, Favaloro UniversityBuenos Aires, Argentina; ^3^National Scientific and Technical Research Council (CONICET)Buenos Aires, Argentina; ^4^Intellectus Memory and Cognition Center, Mental Health and Psychiatry Department, San Ignacio Hospital, Aging Institute, Pontifical Javeriana UniversityBogotá, Colombia; ^5^Laboratory of Cognitive and Social Neuroscience (LaNCyS), UDP-INECO Foundation Core on Neuroscience (UIFCoN), Diego Portales UniversitySantiago, Chile

**Keywords:** PNFA, bvFTD, theory of mind, voxel-based morphometry, social context network model, fronto-insulo-temporal network

## Abstract

Social cognition impairments are pervasive in the frontotemporal dementias (FTD). These deficits would be triggered by (a) basic emotion and face recognition processes as well as by (b) higher level social cognition (e.g., theory of mind, ToM). Both emotional processing and social cognition impairments have been previously reported in the behavioral variant of FTD (bvFTD) and also in other versions of FTDs, including primary progressive aphasia. However, no neuroanatomic comparison between different FTD variants has been performed. We report selective behavioral impairments of face recognition, emotion recognition, and ToM in patients with bvFTD and progressive non-fluent aphasia (PNFA) when compared to controls. Voxel-based morphometry (VBM) shows a classical impairment of mainly orbitofrontal (OFC), anterior cingulate (ACC), insula and lateral temporal cortices in patients. Comparative analysis of regional gray matter related to social cognition deficits (VBM) reveals a differential pattern of fronto-insulo-temporal atrophy in bvFTD and an insulo-temporal involvement in PNFA group. Results suggest that in spite of similar social cognition impairments reported in bvFTD and PNFA, the former represents an inherent ToM affectation whereas in the PNFA these deficits could be related to more basic processes of face and emotion recognition. These results are interpreted in the frame of the fronto-insulo-temporal social context network model (SCNM).

## Introduction

Frontotemporal dementia (FTD) is a neurodegenerative disease commonly characterized by behavioral changes, as well as emotional and cognitive impairments (Neary et al., 1998; Perry and Miller, 2001; Ritchie and Lovestone, 2002). These symptoms present differently in each variant of FTD, namely the behavioral variant FTD (bvFTD), semantic dementia (SD), and progressive non-fluent aphasia (PNFA). Here we compare the structural neuroimaging of social cognition impairments in the bvFTD and PNFA variants.

Facial information (Baron-Cohen et al., 1997; Flombaum and Santos, 2005; Itier and Batty, 2009) and emotion recognition (Sollberger et al., 2010; Shany-Ur et al., 2012; Shany-Ur and Rankin, 2011) are crucial for social cognition (Ibanez et al., 2013a,b). Together with agency perception, they allow for the emergence of inferences about others' affective and cognitive mental states (theory of mind, ToM; Baron-Cohen et al., 1997).

Impaired social cognition and ToM have been shown in bvFTD (Rankin et al., 2003; Torralva et al., 2007, 2009; Manes et al., 2011). Voxel-based morphometry (VBM) studies in bvFTD patients have related those deficits to specific cortical and subcortical atrophy (Eslinger et al., 2007, 2011; Rankin et al., 2009). Furthermore, bvFTD is generally associated with atrophy of the frontal lobe (medial prefrontal, gyrus rectus, orbitofrontal/subgeneual cortices), amygdale, insula, right temporal pole, and white matter tracts, including the anterior corpus callosum, uncinate and arcuate fasiculus, and superior and inferior longitudinal fasciculi (Rosen et al., 2002a; Seeley, 2008; Whitwell et al., 2009; Hornberger et al., 2011; Agosta et al., 2012; Mendez and Shapira, 2013). These areas are engaged in emotion and affective states (Piguet et al., 2011) and also in the social cognition networks (Ibáñez and Manes, 2012; Couto et al., 2013a).

Emotion processing deficits have been also reported in SD (Rosen et al., 2004) and patients suffering from linguistic variants of FTD appear to have deficits in negative emotions (Rosen et al., 2004; Omar et al., 2011). However, no VBM analysis of ToM assessment in PNFA has been reported. This temporal variant of FTD is characterized by left perisylvian, insular and temporal atrophy (Rosen et al., 2002b; Gorno-Tempini et al., 2004; Whitwell et al., 2005; Mesulam et al., 2009).

There are some studies on the morphometry of PNFA related to deficits in emotion recognition (Kumfor et al., 2011; Rohrer et al., 2012; Zhang et al., 2013). However, no previous report has compared the specific atrophy of bvFTD and PNFA related to ToM and none has shown the differences between these samples in the domains of face recognition, basic emotion processing and their interaction with social cognition. Consequently, more research needs to be conducted in this area.

In this study, we describe the differential patterns of brain atrophy that are associated with face recognition, emotional processing, and ToM deficits in a sample of PNFA in comparison with bvFTD patients and control subjects. First, we assessed the profiles of behavioral performance on social cognition. We also compared the atrophy patterns in both FTD groups regarding controls. In addition, a VBM analysis was done on *a priori* selected regions of interest associated with social cognition impairments in FTD (Ibáñez and Manes, 2012), including the orbitofrontal (OFC), insular, amygdaline areas, and temporal pole. The identification of possible deterioration of this network in PNFA would help to clarify whether similar neural correlates for ToM are shared in both FTD groups.

## Materials and methods

### Participants

We recruited 22 patients who fulfilled criteria for diagnoses of behavioral variant of FTD (*n* = 12, 45% male; mean age = 69.81, *SD* = 7.35; mean years of education = 16, *SD* = 6.51) and PNFA (*n* = 10, 55% male; mean age = 64.9, *SD* = 8.68; mean years of education = 12.3, *SD* = 4.49). All patients were in early (mild) stages of the disease.

Patients were assessed and diagnosis was initially made by two experts in FTD (Patricia Montañés and Facundo Manes). Each patient was reviewed in the context of a multidisciplinary clinical meeting, where cognitive neurologists, psychiatrists, and neuropsychologists discussed each patient's case under the current criteria (Gorno-Tempini et al., 2011; Rascovsky et al., 2011). All FTD patients were recruited as part of a broader ongoing study on fronto-temporal dementia and underwent a standard examination battery including neurological, neuropsychiatric, and neuropsychological examinations, and in a separate session, a MRI. They all showed frontal or temporal atrophy on MRI, hence, they belong to probable FTD under the new diagnostic criteria (Gorno-Tempini et al., 2011). Inter-rater reliability for diagnosis was assessed (Cohen's κ = 0.91). The patients described in the present study presented with prominent changes in personality social behavior verified by a caregiver and did not meet criteria for any other psychiatric disorder.

Two groups of controls were assessed. Eighteen healthy controls were recruited for the behavioral assessment and were matched in age (mean, 6.24; *SD*, 7.24), gender (70% male) and years of education (mean, 14.5; *SD*, 3.71; see Table 1). They were recruited from a large pool of volunteers who did not have a history of drug abuse or a family history of neurodegenerative or psychiatric disorders. A second group of 12 healthy controls, age (mean, 60.63; *SD*, 4.59) and education (mean, 15.5; *SD*, 3.13) matched were scanned with a structural MRI to be compared with patients (See Table 1). All participants provided written informed consent in agreement with the declaration of Helsinki and the institution's ethics committee approved the study.

**Table 1:** **Demographic data and results of cognitive state and language assessments**.

	**bvFTD (*n* = 12)**	**PNFA (*n* = 10)**	**Controls (*n* = 18)**	**bvFTD vs. Controls**	**PNFA vs. Controls**	**bvFTD vs. PNFA**
**DEMOGRAPHIC DATA**						
Age (years)	69.8 (7.3)	64.9 (8.6)	69.8 (7.3)	N.S	N.S	N.S
Gender (F:M)	(5:7)	(4:6)	(6:12)	N.S	N.S	N.S
Education (years)	16.0 (6.5)	12.3 (4.4)	14.7 (3.7)	N.S	N.S	N.S
**GENERAL COGNITIVE STATE AND LANGUAGE**						
MMSE	21.7 (8.2)	21.1 (6.2)	29.4 (0.6)	0.002	0.001	N.S
Naming	48.7 (7.3)	29.5 (19.1)	57.6 (3.6)	0.05	0.0001	0.02
Phonological fluency	6.4 (3.6)	7.8 (2.4)	14.5 (4.9)	0.001	0.004	N.S
Semantic fluency	5.5 (3.9)	8.8 (3.9)	16.3 (3.0)	0.0001	0.0002	N.S

*Note: The results are shown as the mean (SD). MMSE, Mini-mental state examination; bvFTD, behavioral variant FTD; PNFA, progressive non-fluent aphasia*.

### Behavioral assessment

Patients and behavioral controls' sample received a series of tasks previously reported by Torralva et al. (2009) that were designed to assess face recognition, facial emotion recognition, and ToM (Reading the mind in the eyes test, RMET; Baron-Cohen et al., 1997).

#### Face recognition

This test assesses the ability to discriminate permanent facial features and a person's identity. The task consists of 10 sheets, each one with 7 black and white pictures of faces of people of different age and gender, who are not facing the camera. The target picture is located at the top of each sheet and is repeated among other six options located at the bottom of the sheet. Participants are asked to match the target face with the option that corresponds to the same person. Performance was expressed as the number of correct responses out of the total, 10.

#### Facial emotion recognition

The task consists of a sheet with cartoons showing the face of a child with 8 different expressions including 5 different basic emotions (anger, sadness, surprise, fear, and happiness). The three remaining expressions were envy, rage, and crying. Participants were asked to identify which one of these pictures corresponded to the expression that the examiner indicated. Negative emotions were grouped in a single global score for VBM correlations. The total score (8) was derived from the number of correct responses.

#### Reading the mind in the eyes test (RMET)

This test (Baron-Cohen et al., 1997) assesses the ToM's emotional inference. This is a computerized test of 17 pictures of the eye region. Participants were asked to choose which of four words best described what the person was thinking or feeling in each photograph. The total score (17) was derived from the number of correct responses.

#### Imaging recordings

Both groups of patients (bvFTD and PNFA) and 12 controls participants were scanned in a 1.5 T Phillips Intera scanner with a standard head coil. A T1-weighted spin echo sequence was used to generate 120 contiguous axial slices (*TR* = 2300 ms; *TE* = 13 ms; flip angle = 68°; FOV = rectangular 256 mm; matrix size = 256 × 240 × 120; slice thickness = 1 mm) which covered all the brain surface and tissue.

#### Voxel-based morphometry (VBM)

Images were preprocessed for VBM analysis using DARTEL Toolbox and following procedures previously described (Ashburner and Friston, 2000). Following, modulated, 12 mm full-width half-maximum kernel smoothed as suggested in other reports (Good et al., 2001) and normalized to MNI space, images were analyzed within general linear models in SPM-8 2nd level analyses (http://www.fil.ion.ucl.ac.uk/spm/software/spm8).

First, a two-sample *t*-test between controls and the whole FTD group was performed in order to account for global atrophy pattern in patients, correcting by total intracranial volume. Second, we performed region of interest (ROI) analyses based on a fronto-insulo-temporal network to describe differential patterns of atrophy on these areas related to the social-context network (Ibáñez and Manes, 2012). The differential patterns of fronto-insulo-temporal atrophy associated with each specific behavioral impairment (face recognition, emotion, and ToM scores separately) in PNFA relative to bvFTD, were evaluated by testing the interaction terms in three different SPM regression designs. This has been shown to be powerful for assessing between-group differences in brain-behavior association slopes when adjusting for interaction terms which in our case, was intracranial volume (O'Brien et al., 2011). Following previous reports, statistical threshold was set at the *p* < 0.05 voxel level, besides small-volume corrected (Grossman et al., 2004) and cluster size corrected (Forman et al., 1995). The GM ROIs were defined *a priori* using the WFU-Pick Atlas (http://www.nitrc.org/projects/wfupickatlas/) in SPM8. Selected ROIs were lateral and medial OFC (Brodmann Area, BA 47/11), gyrus rectus (BA 11), fusiform gyrus (BA 37), bilateral temporal pole (BA 37–38), bilateral amygdale and bilateral insula (BA 13). It should be noted that correlation scatter plots of all tests reported here were assessed for the presence of outliers which may have affected the results, which revealed no outliers.

## Results

### General cognitive status and language assessment

Compared with behavioral control sample, patients had no significant differences in age [*F*_(2, 36)_ = 1.16, *p* = 0.32], gender [*X*^2^_(2)_ Kruskal–Wallis = 1.29, *p* = 0.25], or education [*F*_(2, 36)_ = 1.36, *p* = 0.27]. Similarly, regarding MRI control sample, there were no significant differences in age [*F*_(2, 36)_ = 0.27, *p* = 0.76], gender [*X*^2^_(2)_ Kruskal–Wallis = 6.41, *p* = 0.72], nor in education [*F*_(2, 36)_ = 2.10, *p* = 0.14]. See Table 1.

As expected, differences among bvFTD and PNFA groups [*F*_(2, 36)_ = 10.19, *p* < 0.01] were observed in MMSE total score. *Post-hoc* bivariate comparisons (Tukey HSD, *MS* = 30.74, *df* = 36) showed that both bvFTD (*p* < 0.01) and PNFA (*p* < 0.01) patients had lower scores than controls. Furthermore, the performance on the naming task differed among groups [*F*_(2, 36)_ = 21.15, *p* < 0.01]. *Post-hoc* analysis (Tukey HSD, *MS* = 103.85, *df* = 36) evidenced that both patient groups, bvFTD (*p* < 0.01) and PNFA (*p* < 0.01) showed impairments compared to controls, and that PNFA scored lower than the bvFTD group (*p* < 0.01). Differences among groups were also observed in the phonological fluency task [*F*_(2, 36)_ = 17, *p* < 0.01]. According to *post-hoc* analysis (Tukey HSD, *MS* = 17.50, *df* = 0 36), both bvFTD (*p* < 0.01) and PNFA (*p* < 0.01) performed lower than controls' group. Finally, groups also differed on the semantic fluency task performance [*F*_(2, 36)_ = 33.14, *p* < 0.01]. *Post-hoc* comparisons (Tukey HSD, *MS* = 12.31, *df* = 36) revealed that both bvFTD (*p* < 0.01) and PNFA (*p* < 0.01) patients exhibited lower fluency than controls (see Table 1).

### Behavioral results

**Figure 1:**
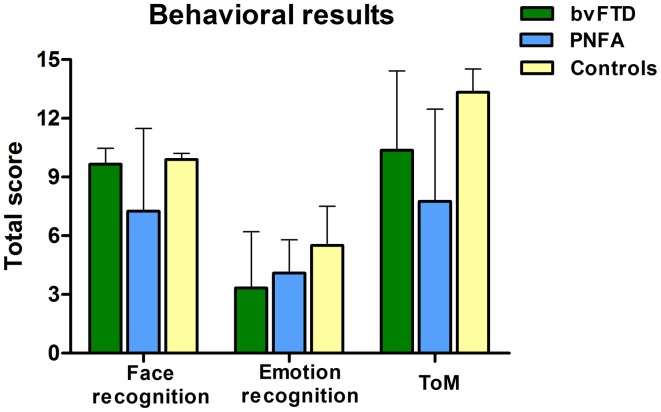
**Scores of three behavioral tasks of face and emotion recognition and ToM.** PNFA patients in light blue, bvFTD patients in green, and controls in beige.

Compared to controls, the bvFTD were not impaired on the face recognition task (*t* = 1.18, *p* = 0.24). However, bvFTD showed impairment in total emotion recognition score (*t* = 2.28, *p* = 0.03), and ToM (*t* = 2.93 *p* < 0.01) in comparison to normal subjects. The PNFA group showed significant impairments in face recognition (*t* = 2.69, *p* = 0.01). Furthermore, they also showed a trend to misrecognize emotions (*t* = 1.93, *p* = 0.06) and a significant deficit in ToM (*t* = 4.80, *p* < 0.001) when compared to healthy subjects (See Figure 1 and Table 2).

**Table 2:** **Behavioral results descriptive and difference statistics**.

	**bvFTD (*n* = 12)**	**PNFA (*n* = 10)**	**Controls (*n* = 18)**	**bvFTD vs. Controls**	**PNFA vs. Controls**	**bvFTD vs. PNFA**
Face recognition	9.6 (0.8)	7.2 (4.2)	9.8 (0.3)	N.S	0.01	N.S
Emotion recognition	3.3 (2.8)	4 (1.7)	5.5 (2.0)	0.03	0.06	N.S
Theory of mind	10.3 (4.0)	7.7 (4.7)	13.3 (1.1)	0.006	<0.001	N.S

*The results are shown as the mean (SD). bvFTD, behavioral variant FTD; PNFA, progressive non-fluent aphasia. VBM, voxel-based morphometry, bvFTD, behavioral variant FTD; PNFA, progressive non-fluent aphasia; BA, Brodman Area; IFG, inferior frontal gyrus; OFC, orbitofrontal cortex*.

### VBM results

#### Global atrophy of patients compared to controls

**Figure 2:**
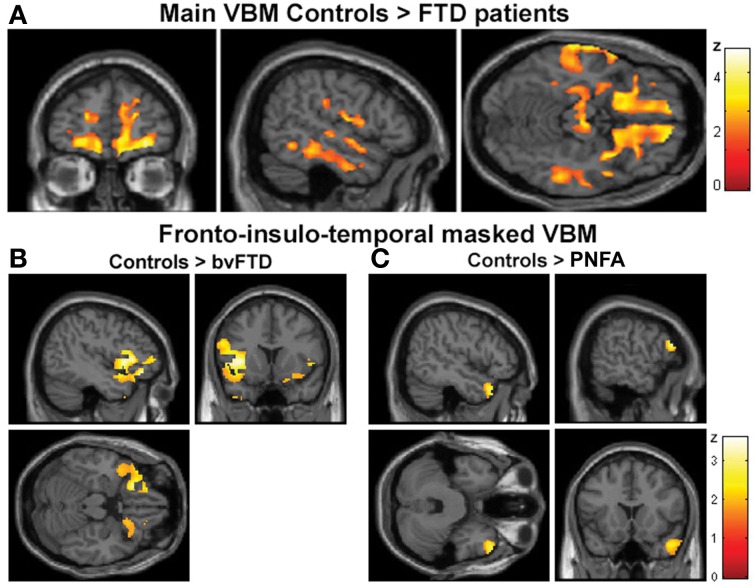
**(A)** Main VBM results, atrophy of all FTD patients compared to controls, *p* < 0.05, α = 0.2 FDR corrected. **(B)** Atrophy of bvFTD group compared to controls in the frontal, insular, and temporal regions, *p* < 0.05, small volume corrected. **(C)** Atrophy of PNFA group compared to controls in the frontal, insular, and temporal regions, *p* < 0.05, small volume corrected.

The VBM analysis revealed a pattern of global atrophy in the FTD group (both versions) in frontal and temporal lobe structures, as expected from and reported in previous studies (Rankin et al., 2006; Seeley et al., 2009; Garibotto et al., 2011). These included orbital (OFC) and motor cortices, right superior and mid temporal gyri, bilateral insula, right anterior cingulate cortex (ACC) and left parietal cortex (*p* < 0.05, α = 0.2 FDR corrected. See Figure 2A and Table 3A).

Table 3**VBM results of whole brain atrophy of patients**.

**Table:** 

**Region**	**BA**	**Coordinates**			***Z*-peak**
		***x***	***y***	***z***	
(A) VBM MAIN EFFECT^*^						
Superior Frontal Gyrus L	6	−22.62	0.73	60.02	1.69
Inferior Frontal Gyrus R	44	56.24	7.19	21	3.42
Precentral gyrus L	4	−62	−7.5	22.5	1.93
Supplementary Motor Area R	6/9	66	9	13.5	3.14
Supplementary Motor Area L	6/9	−15	0.73	63.9	2.81
Anterior Cingulate R	32	5.82	39.5	0	3.01
Frontal Superior Medial R	8	7.11	45.94	45.80	2.49
Frontal Inferior Triangular L	8	−9	36.90	57.43	2.37
Frontal Superior Orbital R	11/47	13.57	42.06	−18.85	2.65
Frontal Superior Orbital L	11/47	−34.26	30.79	−21.43	1.79
Rectus gyrus R	11	3	22.5	−22.5	2.34
Rectus gyrus L	11	−4.5	49.5	−22.5	2.07
Insula R	13	40.7	18	−11	2.14
Insula L	13	−31.67	21.8	3	1.83
Superior Temporal Gyrus R	22	67.5	−21	−1.5	2.93
Temporal Mid gyrus R	21	51	25.10	−4.63	1.73
Inferior Temporal Gyrus R	20	65.29	−36.73	−24	2.07
Parahippocampal Gyrus L	–	−29	−25.10	−16.50	2.03
Fusiform Gyrus L	37	−37.5	−69	−18	4.78
Postcentral gyrus L	3. 1. 2	−60	−2252	28	2.56
Supramarginal gyrus L	40	−60	−21.23	39.33	3.32

**Table:** 

(B) SPECIFIC bvFTD AND PNFA ATROPHY^**^						
**bvFTD**	Anterior insula R	13	45.90	7.19	−2.04	2.58
	Anterior insula L	13	−40.73	9.77	−4.63	2.69
	Lateral OFC R	47	45.90	36.90	−12.38	1.85
	Lateral OFC L	47	−49.77	27.85	−12.38	2.56
	Temporal pole sup L	38	47.19	11.06	−43.41	1.97
	Temporal pole mid R	38	−37.17	10.09	−43.09	2.35
**PNFA**	Temporal pole mid R	38	52.36	18.81	−29.19	2.73
	IFG L	45/46	−56.24	26.56	16.06	2.68

* p < 0.05, FDR corrected α = 0.2;

** *p < 0.05, Small volume corrected*.

Furthermore, regional analysis of the fronto-insulo-temporal sites in the bvFTD group relative to controls showed atrophy in bilateral anterior insula, lateral OFC bilaterally, left inferior frontal gyrus (IFG), left superior and right mid temporal pole regions (*p* < 0.05, small volume corrected, see Figure 2B). On the other hand, the same procedure revealed diminished GM in the PNFA group in right mid temporal pole and left IFG (*p* < 0.05, small volume corrected. See Figure 2C and Table 3B).

In sum, main GM loss was evidenced for FTD patients mainly in OFC, ACC, insula, and lateral temporal cortices. In addition, regional small volume corrected atrophy was greater for bvFTD, in frontal inferior, bilateral insula, and right temporal pole than for PNFA, where only left IFG and temporal pole atrophy was observed.

#### Relative atrophy of PNFA to bvFTD associated to face recognition, emotion, and social cognition

**Figure 3:**
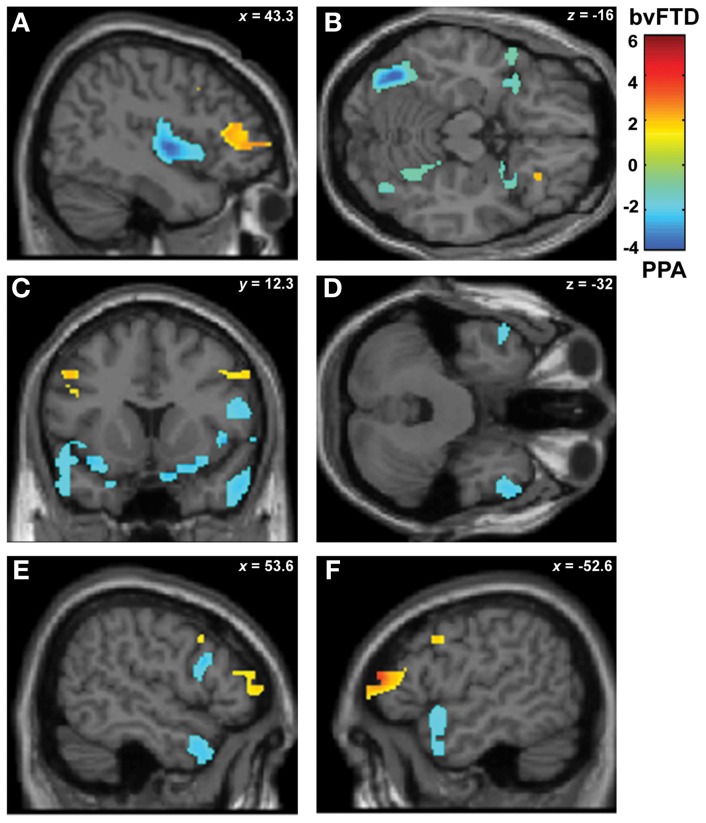
**Contrast vectors of bvFTD (red-yellow) and PNFA (light blue) atrophy that correlates with face recognition.** *p* < 0.05, small volume corrected. **(A)** Right insular (PNFA) and IFG (bvFTD) correlations; **(B)** Bilateral posterior fusiform and temporal pole correlations for PNFA; **(C)** Bilateral insular and temporal pole (PNFA) and mid frontal gyrus (bvFTD) correlations; **(D)** Bilateral temporal pole in PNFA; **(E)** and **(F)**. Most lateral right **(E)** and left **(F)** frontal and temporal pole associations for PNFA and bvFTD. PNFA, progressive non-fluent aphasia, bvFTD, behavioral variant FTD; IFG, inferior frontal gyrus.

In a second stage, the specific engagement of the fronto-insulo-temporal network on emotions and social cognition was assessed through ROI analyses. Face recognition in bvFTD was associated with GM *decreasing* in bilateral OFC (*r* = 0.45; *p* < 0.05), IFG (*r* = 0.19; *p* < 0.05), right gyrus rectus (*r* = 0.36; *p* < 0.05), and right insula (*r* = 0.45; *p* < 0.05). Bilateral fusiform gyrus involvement (*r* = 0.56; *p* < 0.05), temporal pole (*r* = 0.55; *p* < 0.05), insula (*r* = 0.35; *p* < 0.05) and IFG (*r* = 0.46; *p* < 0.05) were observed in PNFA (see Figures 3A–F and Table 4A).

**Table 4:** **Patterns of trophy correlated with task performance in bvFTD and PNFA**.

**Regional GM-task**	**Region**	**BA**	**Coordinates**			***Z*-peak**
			***x***	***y***	***z***	
**(A) FACE RECOGNITION**						
bvFTD > PNFA	Inferior frontal (IFG) R	45	39	33	12	3.22
	Orbitofrontal	11/47	21.33	16.67	−21.43	2.17
		11/47	−34.26	30.79	−21.43	1.79
PNFA > bvFTD	Insula R	13	49.77	1.26	0	3.22
			40.7	18	−11	2.14
	Insula L		−31.67	21.8	3	1.83
	Fusiform L	37	−37.5	−69	−18	4.78
	Fusiform R	37	42	−66	−18	2.25
	Temporal Pole L	38	−53.65	12.35	−16.26	1.85
	Temporal Pole R	38	56.24	11	−29.19	2.32
	Inferior frontal (IFG) R	45	55.5	12.35	17.35	2.10
**(B) EMOTION RECOGNITION**						
bvFTD > PNFA	Rectus	11	−4.5	22.5	22.5	2.34
		11	3	49.5	−22.5	2.07
	Orbitofrontal R	11/47	15	57	−13.5	3.28
	Insula R	13	45.9	−17.35	16	1.82
PNFA > bvFTD	Insula	13	43	−12	7	3.07
		13	−38.14	7.19	−2.04	4.67
	Temporal Pole	38	−31.68	5.9	−22.72	2.15
		38	35.55	6	−22.5	2.95
	Amygdale	−	−25.21	2.02	−18.85	2.95
		−	35.55	6	−22.5	2.95
	Rolandic Opercula L	46	−47.19	4.6	12.18	1.92
**(C) ToM**						
bvFTD > PNFA	Rectus R	11	5	50	−22	1.98
PNFA > bvFTD	Insula	13	40.7	18	−11	1.83
		13	−38.14	11.06	−4.5	2.42
	Amygdale	−	−26.5	4.6	−18.85	2.86
		−	25.21	0.73	−19.5	2.44
	Temporal pole	38	−40.73	3.31	−17.55	2.31
		38	34	11	8	1.99

*VBM, voxel-based morphometry; BA, Brodmann area; PNFA, progressive non-fluent aphasia; bvFTD, behavioral variant-FTD; ToM, theory of mind; R, rigth; L, left; GM, gray matter*.

**Figure 4:**
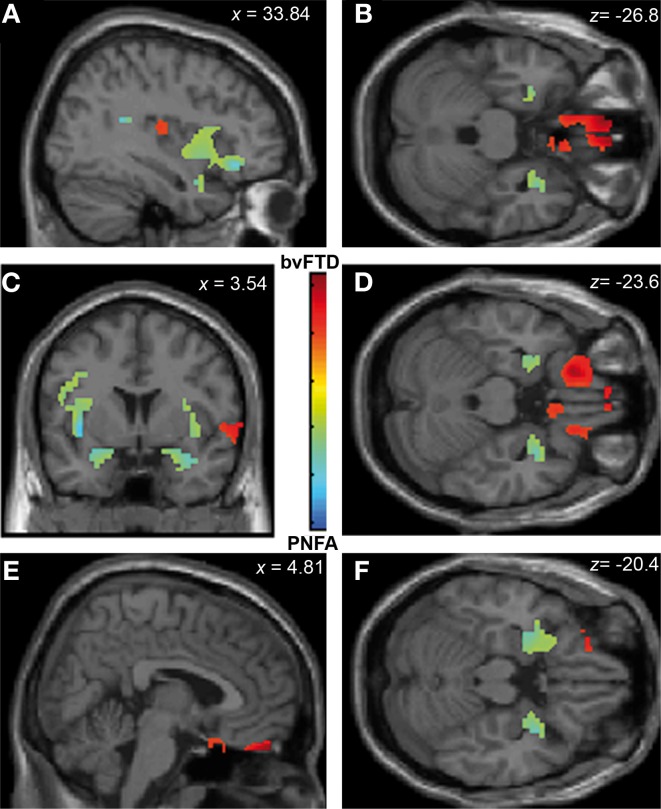
**Contrast vectors of bvFTD (red-yellow) and PNFA (light blue) atrophy correlated with emotion recognition.** *p* < 0.05, small volume corrected. **(A)** Right insular and temporal correlations; **(B,D,F)** Orbitofrontal and mid temporal regions; **(C)** Bilateral insular correlations of PNFA emotions; **(E)** Right gyrus rectus for bvFTD. PNFA, progressive non-fluid aphasia, bvFTD, behavioral variant FTD.

Emotion recognition in bvFTD correlated with bilateral OFC (*r* = 0.74; *p* < 0.05), right gyrus rectus (*r* = 0.46; *p* < 0.05), and right insula (*r* = 0.26; *p* < 0.05). In PNFA, emotion recognition was associated with atrophy of bilateral insula (*r* = 0.38; *p* < 0.05), temporal pole (*r* = 0.72; *p* < 0.05) bilateral amygdala (*r* = 0.23; *p* < 0.05) and left rolandic opercula (*r* = 0.62; *p* < 0.05; see Figures 4A–F; Table 4B). The recognition of negative emotions correlated with bilateral OFC (*r* = 0.51; *p* < 0.05), and fronto-insular cortex (FIC, *r* = 0.66; *p* < 0.05) and right gyrus rectus (*r* = 0.44; *p* < 0.05) in bvFTD; and in PNFA with right insula (*r* = 0.72; *p* < 0.05) and right temporal pole (*r* = 0.50; *p* < 0.05).

**Figure 5:**
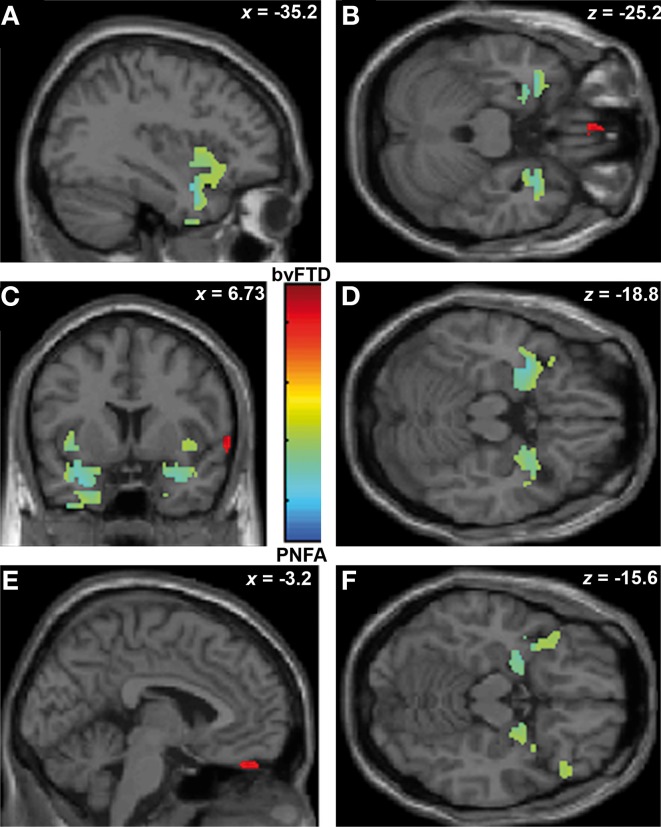
**Contrast vectors of bvFTD (red-yellow) and PNFA (light blue) atrophy that correlates with ToM.** *p* < 0.05, small volume corrected. **(A)** Right insulo-temporal correlations for PNFA; **(B,D,F)** Temporal pole and amygdaline correlations for PNFA; **(C)** Bilateral insular correlations of PNFA; **(E)** Right gyrus rectus for bvFTD. PNFA, progressive non-fluid aphasia, bvFTD, behavioral variant FTD.

Finally, ToM correlated with gyrus rectus (*r* = 0.49; *p* < 0.05) in the bvFTD group; and with bilateral insula (*r* = 0.54; *p* < 0.05), temporal pole (*r* = 0.36; *p* < 0.05) and amygdale (*r* = 0.36; *p* < 0.05) in the PNFA group (see Figures 5A–E and Table 4C).

Summarizing, face recognition was associated in bvFTD with atrophy in bilateral orbitofrontal regions, and right rectus gyrus, while in PNFA face recognition was related to right insula and bilateral fusiform gyrus. Emotion deficits were associated to relative atrophy of FIC (bvFTD) and of right temporal pole plus bilateral insula (PNFA), whereas ToM impairments correlated with fronto-insular areas (bvFTD) and with right insula and temporal pole (PNFA; See Figures 3–5; and Table 4).

## Discussion

This work compared the structural neuroimaging signatures associated with face recognition, emotional processing and ToM impairments in bvFTD and PNFA. Compared to controls, impairments in face recognition (PNFA), emotion recognition and ToM (PNFA and bvFTD) were observed. VBM analysis showed the expected atrophy of orbital, medial, and lateral frontal structures, insula, temporal, and parietal cortices in both FTD versions compared to age and gender matched healthy controls. Furthermore, behavioral deficits were associated with different patterns of atrophy in each FTD variant. ROI analysis looking at the fronto-insulo-temporal network in bvFTD revealed a pattern of bilateral orbitofrontal and gyrus rectus, right fronto-insula and insula associated with emotion and social cognition deficits respectively, whereas in PNFA these impairments were related to right insula and right temporal pole atrophy. This is the first report unraveling the structural correlates of face recognition, emotion, and ToM in both variants of FTD. We discuss these findings in the light of relatively differential (low level) emotion-face recognition processes vs. (higher level) social cognition impairments in these disorders.

### Atrophy patterns in PNFA and bvFTD and their relation with emotion/social cognition performance

The general pattern of atrophy found in our FTD patients described above is in coincidence with previous morphometric evidence and distinct methodological techniques (Seeley et al., 2008; Zamboni et al., 2008; Rohrer et al., 2010). Moreover, functional and structural connectivity studies have shown atrophy in similar regions (Seeley, 2008; Zhou et al., 2010; Agosta et al., 2012; Mendez and Shapira, 2013) although the structural correlates of face recognition, emotion recognition, and ToM in both groups reveal different levels of social cognition impairments.

#### Face recognition

When analyzing the bvFTD fronto-insulo-temporal pattern of atrophy, face recognition performance correlated with orbitofrontal cortex. This region is associated with covert face recognition in prosopagnosia (Valdes-Sosa et al., 2011), with visual encoding of face stimuli (Frey and Petrides, 2003; Henson et al., 2003) and with familiar faces (Taylor et al., 2009). Hence, in bvFTD, OFC can be traced as a region subserving face perception.

On the other hand, face recognition impairments in PNFA were related to atrophy of bilateral posterior fusiform gyrus, bilateral insular cortex, and anterior temporal lobe (ATL). Specifically, atrophy of the posterior fusiform is not an unexpected result, since the Fusiform Face Area (FFA) selectively engaged on early stages of face recognition, was initially described by Kanwisher et al. (1997) less than 10 mm further from our PNFA atrophy peak (MNI *x, y, z* coordinates, left: −35.35 −64.35 −15.63; right: 40.40 −56.11 −15.16, see Table 4 to compare with atrophy coordinates). On the other hand, an influential macaque study (Freiwald and Tsao, 2010) describes a network for face recognition with its ATL patches located in ventral and superior temporal pole as well as in the anterior bank of the STS, which has been recently confirmed in humans by a combination of fMRI meta-analytic and empirical results (Von Der Heide et al., 2013). In addition, a recent review by Gainotti (2007) shows that patients with right temporal pole damage are more prone to familiar face recognition deficits and poorer naming from facial (visual) stimuli than those who have left temporal pole lesions. This work also points to models of continuity between multimodal perceptual features and conceptual activities, leading to the emergence of familiarity feelings (Bruce and Young, 1986). In line with this wealth of evidence, we found a pattern of posterior fusiform gyrus and right temporal pole atrophy associated to face recognition scores in PNFA that would suggest an engagement of both early discriminative and person-specific stages of face recognition and supports their role in indexing semantic/biographical knowledge (Zahn et al., 2007; Mion et al., 2010; Ross and Olson, 2010; Simmons et al., 2010). Nonetheless, the process of face recognition includes the extraction of emotional expression (Haxby et al., 2000), which contributes to familiarity feelings and person perception (Young and Bruce, 2011). In the classical Bruce and Young model (1986), semantic processing is an integral part of the face structural processing which indexes the attribution of meaning, valence, and salience to facial expressions. With respect to this, we found bilateral insular atrophy in PNFA associated to face recognition, which we speculate may be accounting for the conveyed emotional component of this task (Nakamura et al., 2000; Josephs et al., 2008; Nielson et al., 2010). Furthermore, the structural connectivity of anterior insula, temporal pole, and orbitofrontal regions points to a dynamic interaction among these areas in ascribing emotional salience to perceived stimuli (Cloutman et al., 2012). Hence, we propose that the neuroanatomical correlates of PNFA's face recognition would engage both basic face recognition and emotional processing areas.

#### Emotion recognition

In bvFTD, the fronto-insulo-temporal pattern of atrophy that correlated with emotion recognition included orbitofrontal, gyrus rectus and right posterior insula atrophy, regions which have been known to participate in assigning emotional valence to facial stimuli (Gobbini and Haxby, 2007). In addition, these emotion recognition deficits in PNFA involved some of the same regions related to face recognition deficits (insula, right temporal pole, and rolandic opercula). These are structures engaged in the binding of perception and visceral emotional responses (Olson et al., 2013; Visser et al., 2012). Additionally, we found that emotion recognition deficits in PNFA also related to specific atrophy in the left amygdale, a structure classically engaged in emotional processing (Kennedy and Adolphs, 2012) and previously related to emotion deficits in PNFA and SD (Whitwell et al., 2005; Garibotto et al., 2011; Yang et al., 2012). This corresponds to the temporo-insular (but not frontal) atrophy associated with ToM observed in PNFA, and it leads us to posit that lower level recognition of basic emotions in PNFA together with the face recognition impairments might represent basic deficits which could be triggering ToM deficits in these patients.

#### ToM

Similarly, in bvFTD the ToM deficit was mainly associated with two core regions for social skills such as OFC and gyrus rectus bilaterally. These areas index high level social cognition processes (Viskontas et al., 2007; Nestor et al., 2012). Moreover, previous morphometric reports related OFC atrophy to ToM deficits in bvFTD (Mesulam et al., 2009; Eslinger et al., 2011).

Second, we found that the ToM deficit in PNFA was associated with temporal pole and insular cortex degeneration. These regions are engaged in semantic knowledge and in the integration of emotional body states and external milieu information respectively, two processes closely related to social cognition (Olson et al., 2007; Craig, 2009; Ibanez et al., 2010; Couto et al., 2013b; Melloni et al., 2013). As mentioned above, biographical and emotional knowledge contribute to the recognition of faces. Further evidence suggests that temporal pole atrophy in SD could be related to ToM deficits (Duval et al., 2012) and right temporal pole atrophy has been associated to prosopagnosia in SD (Josephs et al., 2008). Therefore, right temporal pole and insular cortex atrophy in PNFA could be related to ToM deficits as they are associated with specific emotional and face recognition processes crucial for this social domain. This is convergent with the anatomical correlations of face and emotion recognition deficits described above. In other words, we suggest that the lower level emotional and face recognition deficits associated with the right temporal pole and insular atrophy could be the roots of subtle ToM impairments in PNFA, rather than a *sui generis* frontal involvement as is the case of bvFTD (Olson et al., 2007, 2013; Visser et al., 2012).

Although on the basis of these results we cannot rule out the possibility that concomitant loss of executive functions, language, and semantic memory may be the cause of social skills deployment in our PNFA sample, the hypothesis outlined above is also consistent with the multifactorial nature of emotional deficits proposed in FTD (Miller et al., 2012). Indeed, the fact that these recognition impairments impact the processing of social meaningful features on PNFA could be considered an extension of the semantic phenotype to the social and emotional domains. This parallels what has been shown to occur in bvFTD, in which the same ToM would be associated with other cognitive domains such as executive functions (Torralva et al., 2009). This has two clinical implications: first, it suggests that different assessment batteries should be designed and applied for targeting divergent cross-domains associations in each variant (Torralva et al., 2009); second, the possible impact of more basic cognitive processes on social domains should be considered for the neuropsychological assessment of PNFA and bvFTD patients. Finally, our results suggest that different cognitive and neuroanatomic pathways would affect ToM performance, in a relationship that is not only restricted to executive functions as reported in many papers, but also extended to basic face recognition and emotion processing (Ibanez et al., 2013a,b).

A limitation of this study is that although they are age, gender, and formal education matched, MRI control subjects were not the same as the ones in which task impairments were compared. Therefore, there is an indirect relationship between patients and controls' brain morphology on the one hand, and specific VBM associations with behavioral impairments, on the other hand. However, as the neural underpinnings of social cognition impairment in bvFTD are well-characterized and a common substrate of atrophy is present in both versions of FTD, we intended here to use bvFTD patients as a comparison sample to reveal the neural correlates of social cognition deficits in PNFA as done in previous reports (Seeley et al., 2008). Hence, we posit this is a more straightforward way of comparing both variants of this neurodegenerative disease. Another limitation is the small sample size which could be susceptible of spurious correlation and may have biased the results of regression analyses. However, several previous studies cited in this paper have used a similar sample size (Eslinger et al., 2007; Seeley et al., 2008; Zamboni et al., 2008).

## Conclusions

In brief, our results suggest that primary face and emotion recognition impairments would impact on ToM in PNFA, whereas in bvFTD the ToM deficits seem to be a sui generis impairment, with preservation of basic face recognition. Both results point to possible existence of alternative pathways to ToM impairment in these conditions. Hence, although not behaviorally dissociable, ToM seems to be dissociable neuroanatomically and this would suggest extended circuits that support this function.

Although relatively similar impaired performance in social cognition was observed in both FTD groups, those similar impairments can be related to different processes and atrophy patterns in PNFA and bvFTD. Particularly, the development of social cognition signatures in PNFA would not be solely related to the typical atrophy of frontal social nodes as in bvFTD. The basic recognition and emotion stages of face recognition impairments in PNFA patients (related to right temporal pole and insular cortex atrophy), could account for the processing of social skills such as ToM. In addition, different regions of the so called social context network model (SCNM; Ibáñez and Manes, 2012) are selectively affected in both FTD groups, suggesting their participation on the dynamic interplay between invariant-specific and context-dependent variables and stimuli.

### Conflict of interest statement

The authors declare that the research was conducted in the absence of any commercial or financial relationships that could be construed as a potential conflict of interest.
